# Sulfated polysaccharides effectively inhibit SARS-CoV-2 in vitro

**DOI:** 10.1038/s41421-020-00192-8

**Published:** 2020-07-24

**Authors:** Paul S. Kwon, Hanseul Oh, Seok-Joon Kwon, Weihua Jin, Fuming Zhang, Keith Fraser, Jung Joo Hong, Robert J. Linhardt, Jonathan S. Dordick

**Affiliations:** 1 Department of Chemical and Biological Engineering, Center for Biotechnology and Interdisciplinary Studies, Rensselaer Polytechnic Institute, Troy, NY USA; 2 Department of Chemistry and Chemical Biology, Rensselaer Polytechnic Institute, Troy, NY USA; 3 National Primate Research Center, Korea Research Institute of Bioscience and Biotechnology, Cheongju, Chungcheongbuk Republic of Korea; 4 College of Biotechnology and Bioengineering, Zhejiang University of Technology, Hangzhou, 310014 China; 5 Department of Biological Sciences, Rensselaer Polytechnic Institute, Troy, NY USA

**Keywords:** Mechanisms of disease, Glycobiology

Dear Editor,

COVID-19, caused by the SARS-CoV-2 virus, has now spread worldwide with catastrophic human and economic impacts and currently has infected over 10 million people and killed over 500,000^1^. In an effort to mitigate disease symptoms and impede viral spread, efforts in vaccine development and drug discovery are being conducted at a rapid pace^2^. Recently, we showed that the well-known anticoagulant heparin has exceptional binding affinity to the spike protein (S-protein) of SARS-CoV-2^3^. The S-protein of SARS-CoV-2 bound more tightly to immobilized heparin (*K*_D_ = ~10^−11^ M) than the S-proteins of either SARS-CoV (*K*_D_ = ~10^−7^ M) or MERS-CoV (*K*_D_ = ~10^-9^ M). However, it is not known whether the tight binding of heparin to the SARS-CoV-2 S-protein translates into potent antiviral activity. In the current study, we evaluated the in vitro antiviral properties of heparin and other closely related polysaccharides to assess the relevance of heparin-related GAGs and other sulfated polysaccharides as part of the pharmacopeia of potential therapeutics that target SARS-CoV-2. Vero-CCL81, which expresses both ACE2 and TMPRSS2^4^, were used for viral replication at high titer^5^ for use in antiviral assays.

Heparin, heparan sulfates, other glycosaminoglycans (GAGs)^3^, and fucoidan and other highly sulfated polysaccharides were screened using surface plasmon resonance (SPR) to measure binding affinity to the SARS-CoV-2 S-protein (Fig. 1a). Briefly, solution competition studies between surface immobilized heparin and other sulfated polysaccharides were evaluated by injecting SARS-CoV-2 S-protein (50 nM) alone or mixed with 1 µM of an indicated polysaccharide in SPR buffer at a flow rate of 30 μL/min. After each run, dissociation and regeneration were performed. For each set of competition experiments, a control experiment (S-protein without polysaccharide) was performed to ensure the surface was fully regenerated. Among the tested polysaccharides, RPI-27 and RPI-28, complex sulfated polysaccharides (fucoidans) extracted from the seaweed *Saccharina japonica*^6^, chemo-enzymatically synthesized trisulfated (TriS) heparin^7^, and unfractionated USP-heparin itself were able to compete with heparin for S-protein binding. We selected these compounds along with a non-anticoagulant low molecular weight heparin (NACH)^8^ for further study (Fig. 1b). The other GAGs including heparan sulfate, the chondroitin sulfates, and keratan sulfate show no competitive binding when compared to the control.

**Fig. 1:**
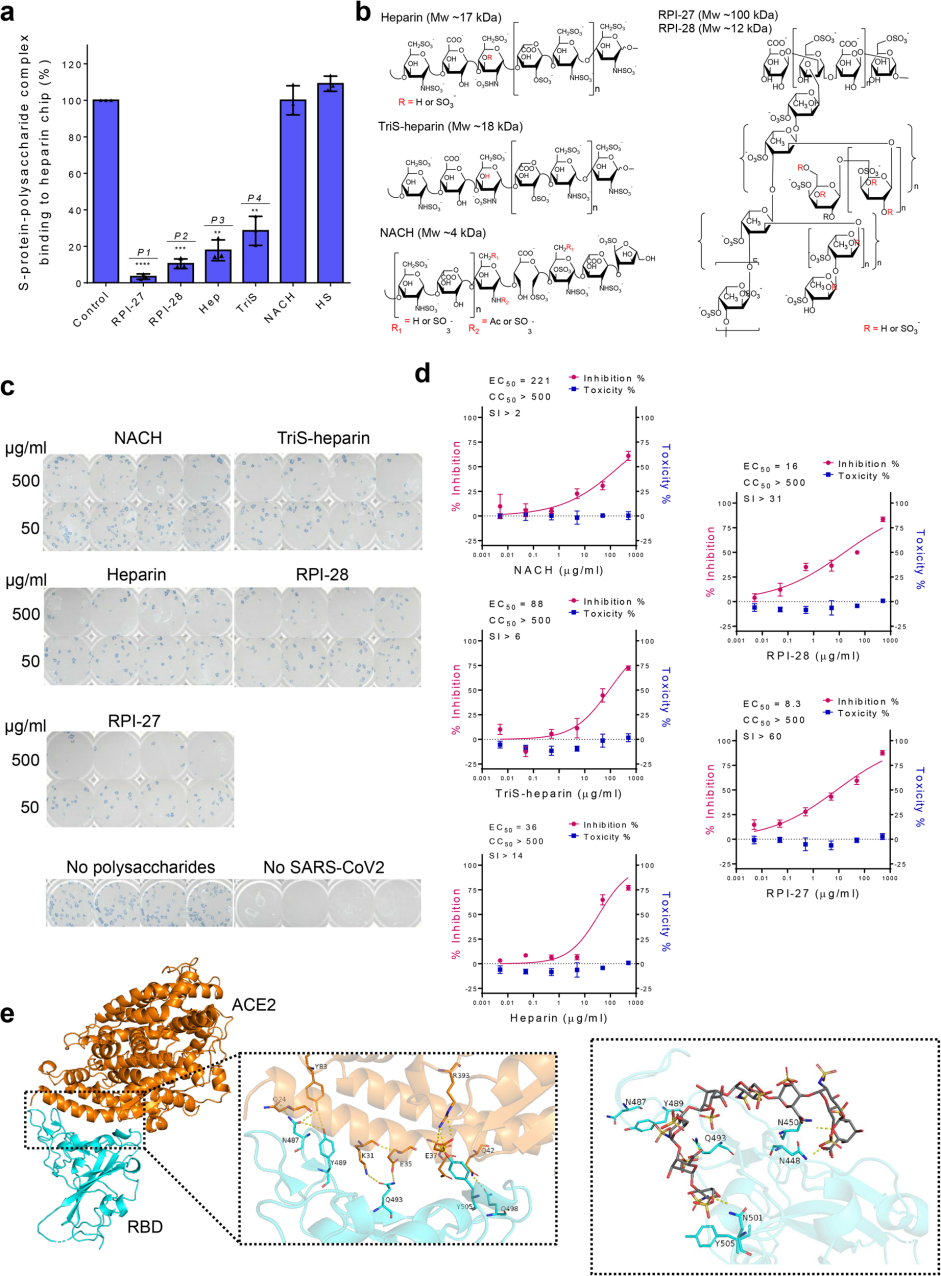
Assessment of antiviral activities of certain sulfated polysaccharides. **a** Surface plasmon resonance (SPR) experiments were used to screen polysaccharides that outcompete immobilized heparin binding to SARS-CoV-2 S-protein. Data are presented as mean±s.d., *n*=3 biologically independent samples. A two-sided *t*-test was performed to test significance against the control (*P1*<0.0001, *P2*=0.0003, *P3*=0.0016, *P4*=0.0041). **b** Structural units comprising polysaccharides used for in vitro antiviral studies. **c** Focus reduction assay images of virus infection on treatment of indicated polysaccharides. At 48h after infection, Vero cells were fixed and probed with SARS-CoV-2 spike primary antibody (1:10000, Sino Bio Inc.) and HRP-conjugated goat rabbit (1:10000, Abcam) secondary antibody. **d** Vero cells were infected with SARS-CoV-2 at a MOI of 2.5×10^−3^ at different doses of each polysaccharide for 48h. The viral yield was quantified using a focus reduction assay. Cytotoxicity in Vero cells was measured using a WST-1 assay. The left and right *y*-axis of the graphs represent mean % inhibition of virus yield and cytotoxicity of the polysaccharides, respectively. Cytotoxicity experiments were performed in duplicate with *n*=3 biologically independent samples. Focus reduction assay experiments were performed in mean±s.d. (quadruplicate measurements) with *n*=3 biologically independent samples. **e** The RBD-ACE2-binding interface is stabilized by an extensive hydrogen bonding network involving sidechains of several residues on both RBD and ACE2. Polar sidechains of N487, Y489, Q493, Q498, and Y505 on the spike protein RBD along with other residues would be able to bind to heparin and inhibit RBD-ACE2 interaction. Heparin (here an octasaccharide) forms a hydrogen bond network with N448, N450, Q493, and N501 that aids in its occupancy of this binding regions and sterically restrict access to Q498, Y489, and Y505 necessary for ACE2 receptor binding.

Standard assays were performed to quantify potential cytotoxicity and antiviral activity. Cytotoxicity determination of the polysaccharides was performed using Vero cells and the standard water-soluble tetrazolium salt-1 (WST-1) assay (Takara Bio Inc., Japan). None of the tested polysaccharides showed toxicity even at the highest concentrations tested. Vero cells were infected with SARS-CoV-2 at a multiplicity of infection (MOI) of 2.5 × 10^−3^ with varying dosages of polysaccharide to confirm antiviral activity. A focus reduction assay was performed 48 h post infection to determine efficacy. Antiviral activities correlated with the SPR results. The most potent compound tested, RPI-27, is a high molecular weight, branched polysaccharide related to the known compound fucoidan, and had an EC_50_ of 8.3 ± 4.6 μg/mL, which corresponds to ~83 nM (Fig. 1c, d and Supplementary Table S1). This is substantially more potent than remdesivir having a reported in vitro EC_50_ value of 770 nM in Vero-E6 cells^9^ and 11.4 µM in Vero-CCL81 cells^10^, currently approved for emergency use for severe COVID-19 infections. The smaller RPI-28 has the same basic structure as RPI-27 but a lower molecular weight and, thus, a lower activity (EC_50_ = 1.2 μM, Supplementary Table S1). Heparin and the TriS-heparin (an intermediate in the bioengineered heparin synthesis pathway^3^) also have potent antiviral activity with EC_50_ values of ~2.1 and 5.0 μM, while the lower molecular weight NACH had an approx. EC_50_ of 55 μM. Similar antiviral activity of heparin has also been demonstrated recently^11^. Heparin and TriS-heparin are similar, with the latter devoid of the relatively small fraction of 3-*O*-sulfate groups present on heparin (Fig. 1b). Thus, their similar activity is expected. However, the low molecular weight NACH had far lower antiviral activity. Less sulfated GAGs, such as heparan sulfate and various chondroitin sulfates, because of their very low S-protein binding were not tested in the antiviral assay.

The high activity of RPI-27 and RPI-28 relative to the other polysaccharides tested may be a result of multivalent interactions between the polysaccharide and viral particle^12^. While heparin, TriS-heparin, and NACH are linear polysaccharides, RPI-27 and RPI-28 are both highly branched (Fig. 1b), possibly conferring added points of interaction in 3-dimensional space. The higher affinity of RPI-27 compared to RPI-28, and hence its more potent antiviral activity, may be due to the far higher molecular weight of the former providing greater opportunity for multipoint binding to the S-protein of SARS-CoV-2. The non-anticoagulant TriS-heparin may be more desirable in some applications than the potent anticoagulant heparin.

Our results reveal that specific sulfated polysaccharides bind tightly to the S-protein of SARS-CoV-2 in vitro, which suggests that they can act as decoys to interfere with S-protein binding to the heparan sulfate co-receptor in host tissues^3,11^, inhibiting viral infection. To model this, we constructed a docking model between heparin and the S-protein receptor-binding site (RBD) using the crystal structure of the chimeric RBD-ACE2 complex (PDB ID: 6VW1)^13^ (Fig. 1e and detailed docking model described in supplementary information). The RBD’s amino acid residues involved in binding the ACE2 (angiotensin-converting enzyme 2) receptor also participated in heparin binding, suggesting a mechanism of viral entry inhibition by heparin. Moreover, the larger the oligosaccharide model used in docking studies, the tighter the binding. Specifically, the octasaccharide binds tighter than the tetrasaccharide (–7.3 vs. –6.1 kcal/mol).

Since these polysaccharides show promising antiviral activity in vitro and low cytotoxicity, we suggest that they may have promising clinical use. Along these lines, SARS-CoV-2 has been found to infect a wide range of tissues that possess sufficient ACE2 levels^14^, including the nose and the gastrointestinal tract^15^. Potential routes of delivery of these non-anticoagulant polysaccharide candidates, including the fucoidans (RPI-27, and RPI-28) and the TriS-heparin, could be through a nasal spray, metered dose inhaler, or oral delivery. This is distinct from remdesivir, which must be delivered intravenously^16^. Indeed, when taken orally, the fucoidans, isolated from edible sulfated seaweed polysaccharides, are considered as “Generally Recognized as Safe” and heparin, an approved drug, is not orally bioavailable. Interestingly, a retrospective clinical study suggests that the administration of anticoagulants, such as heparin, may provide better outcomes for patients hospitalized with COVID-19, including a dramatic reduction in mortality of intubated patients^17^. It is unknown whether this is a result of heparin’s anticoagulation alone, or to some degree is an effect of its anti-SARS-CoV-2 activity. Inhaled heparin has additional benefits such as reducing pulmonary coagulopathy and inflammation without producing systemic bleeding^18^. To this end, we suggest that treatment of fucoidans, nebulized heparin, or possibly TriS-heparin in combination with or without current antiviral therapies, should be assessed first in human primary epithelial cells and then in human patients suffering from COVID-19.

## Supplementary information

Supplementary information, Materials and Table

## Supplementary information

**Supplementary Information** accompanies the paper at (10.1038/s41421-020-00192-8).
