# Supplementary material for: Sulfated polysaccharides effectively inhibit SARS-CoV-2 in vitro
Source: Cell Discov. 2020 Jul 24;6:50. doi: 10.1038/s41421-020-00192-8 (PMC7378085; doi:10.1038/s41421-020-00192-8)
Supplement: Supplementary file 1 — Supplementary information, Materials and Table [file 41421_2020_192_MOESM1_ESM.pdf]

Supplementary Information for:

Sulfated polysaccharides effectively inhibit SARS-CoV-2 *in vitro*

Paul S Kwon[1,5], Hanseul Oh[2], Seok-Joon Kwon[1], Weihua Jin[1,3], Fuming Zhang[1], Keith Fraser[4], Jung Joo Hong[2], Robert J. Linhardt[1,4,5], and Jonathan S Dordick[1,4]

[1]Department of Chemical and Biological Engineering, Center for Biotechnology and Interdisciplinary Studies, Rensselaer Polytechnic Institute, Troy, NY, USA.
[2]National Primate Research Center, Korea Research Institute of Bioscience and Biotechnology, Cheongju, Chungcheongbuk, Republic of Korea.
[3]College of Biotechnology and Bioengineering, Zhejiang University of Technology, Hangzhou, 310014, China
[4]Department of Biological Sciences, Rensselaer Polytechnic Institute, Troy, NY, USA.
[5]Department of Chemistry and Chemical Biology, Rensselaer Polytechnic Institute, Troy, NY, USA

Materials and Methods

Preparation of heparin SPR biochip

S-Proteins: "SARS-CoV2" (MW: 101.7 kDa, Cat: 40591-V02H), coronavirus spike; "SARS-CoV" (MW: 74.4 kDa, Cat: 40150-V08B1); and "MERS-CoV" (MW: 142.2 kDa, Cat: 40069-V08B) were purchased from Sino Biological Inc. Porcine intestinal mucosa heparin (M_W ~17 kDa) was from Celsus Laboratories, Inc. TriS-heparin, M_W ~17.6 kDa, was chemoenzymatically synthesized[3]. NACH, MW ~4 kDa, was chemically synthesized from enoxaparin[4] (Sanofi-Aventis). RPI-27, MW ~100 kDa, and RPI-28, MW ~12 kDa were extracted from seaweed[2]. Sensor SA chips were from GE Healthcare (Uppsala, Sweden). SPR measurements were performed on a BIAcore 3000 operated using BIAcore 3000 control and BIAevaluation software (version 4.0.1).

Biotinylated heparin was prepared by conjugating its reducing end to amine-PEG3-Biotin (Pierce, Rockford, IL). In brief, heparin (2 mg) and amine-PEG3-Biotin (2 mg, Pierce, Rockford, IL) were dissolved in 200 µl H_2O, 10 mg $NaCNBH_3$ was added. The reaction mixture was heated at 70 °C for 24 h, after that a further 10 mg $NaCNBH_3$ was added and the reaction was heated at 70 °C for another 24 h. After cooling to room temperature, the mixture was desalted with the spin column (3,000 MWCO). Biotinylated heparin was collected, freeze-dried and used for SA chip preparation.

The biotinylated heparin was immobilized to streptavidin (SA) chip based on the manufacturer's protocol. The successful immobilization of heparin was confirmed by the observation of a 600-resonance unit (RU) increase on the sensor chip. The control flow cell (FC1) was prepared by 2 min injection with saturated biotin.

The protein samples were diluted in HBS-EP buffer (0.01 M HEPES, 0.15 M NaCl, 3 mM EDTA, 0.005% surfactant P20, pH 7.4). Different dilutions of protein samples were injected at a flow rate of 30 µL/min. At the end of the sample injection, the same buffer was flowed over the sensor surface to facilitate dissociation. After a 3 min dissociation time, the sensor surface was regenerated by injecting with 30 µL of 0.25% sodium dodecyl sulfate (SDS) to get fully regenerated surface. The response was monitored as a function of time (sensorgram) at 25 °C.

Solution competition study between heparin on chip surface and heparin, trisulfated heparin analog, chemically modified heparin or GAGs in solution using SPR

S-protein (50 nM) mixed with 1 µM of heparin, TriS-heparin, NACH, RPI-27, and RPI-28 in HBS-EP buffer were injected over a chip, on which heparin was immobilized, at a flow rate of 30 µL/min, respectively. After each run, the dissociation and the regeneration were

performed as described above.

Virus isolation and propagation

SARS-CoV-2 isolated from Korean patient was provided by the National Culture Collection for Pathogens (NCCP43326). The isolate was propagated in Vero-CCL81 cells (Korean Cell Line Bank, KCLB No. 10081, Korea)[1,2] with Dulbecco's modified Eagle's medium (DMEM), supplemented with 2% FBS, 50 U/ml penicillin, and 50 μg/mL streptomycin. Three days post virus infection, culture supernatants were collected, aliquoted, and stored at -80 ℃. Viral titer was determined by focus formation assay. VERO cells (2 × 10^4 cell/well, 100 μL/well) were infected with a 10-fold serial dilution of SARS-CoV-2 for 1 h and then removed inoculum. Next, 2% carboxymethyl cellulose in 4% FBS in DMEM were overlaid to the each well. Subsequent procedures were conducted under the identical conditions as described below in Section of Focus reduction assay. The average of six wells was calculated as focus forming units (FFU) per ml. All the procedures were performed in a biosafety level 3 laboratory.

Cytotoxicity assay

Water Soluble Tetrazolium Salt-1 (WST-1) assay (Takara Bio Inc., Japan) was performed following the manufacturer's protocol. Briefly, VERO (2 × 10^4 cell/well, 100 μL/well) were seeded in flat-bottom 96-well plates and incubated overnight at 37°C to evaluate the cytotoxicity of polysaccharides on Vero cells. Cells were treated with the different dose of the each polysaccharide (0.5, 0.05, 0.005 mg/ml). After 48 h incubation at 37°C, 10 μL premix WST-1 was added per well and incubated for 1 h at 37°C. Absorbance was measured at 430 nm using ELISA plate reader (Epoch, Bio-Tek Instruments, Inc., USA). The 50% cytotoxic concentration for inhibitors (CC_{50}; polysaccharide concentration that reduced the cell viability by 50% compared to the cell only control) was determined using nonlinear regression analysis (GraphPad Prism 8.4, USA). Each experiment was performed in duplicate wells and repeated three times.

Focus reduction assay

Vero cells (2 × 10^4 cell/well, 100 μL/well) were seeded in 96 well plates to confirm antiviral activity of the polysaccharides. SARS-CoV-2 (multiplicity of infection; MOI = 2.5 × 10^{-3}) was pre-incubated with several doses of the polysaccharide (0.5, 0.05, and 0.005 mg/L for 1 h at 37 °C. Each mixture of virus and polysaccharide was then added to prepared cells to allow infection for 1h. These virus-polysaccharide mixtures were removed and 2% carboxymethyl cellulose in 4% FBS in DMEM were overlaid to the infected cells. The plates

were further cultured at 37°C for 2 days. After incubation, the cells were washed with cold PBS, fixed with 4% paraformaldehyde phosphate buffer solution for 30 min at 4°C, and permeabilized with 0.5 % Triton X-100 for 20 min at room temperature. After washing, the cells were incubated with SARS-CoV-2 Spike antibody (1:10000, Sino Bio Inc.) for 45 min at room temperature. The plates were then washed with 0.05% Tween 20 and then incubated with HRP-conjugated goat rabbit (1:10000, Abcam) for 45 min at room temperature. After repeating the washing, and the cells were stained with 50 µL True Blue peroxidase substrate (KPL) for 20 min in the dark. Subsequently, the plates were washed with tap water and were dried completely. Plate images were captured using an Immunospot CTL reader (S6 Universal analyzer) and the number of foci/well counted. SARS-CoV-2 was tested against each polysaccharide concentration in quadruplicate wells. Experiments were repeated three times. The 50% inhibitory concentration of each polysaccharide against SARS-CoV-2 (IC50; polysaccharide concentration that inhibited FFU formation by 50% compared to the cell only control) was determined using non-linear regression analysis (GraphPad Prism 8.4, USA).

Heparin docking model

Shang *et al* solved the crystal structure (PDB ID: 6VW1) of the molecule that included the RBD (receptor binding domain) of the SARS-CoV-2 spike protein bound to the angiotensin-converting enzyme 2 (ACE2) receptor.[3] The availability of this structure allowed us to generate a model descriptive of the mechanism through which glycosaminoglycans may inhibit spike protein RBD-ACE2 binding. The RBD-ACE2 binding interface is stabilized by an extensive hydrogen bonding network involving sidechains of several residues on both RBD and ACE2. We took advantage of the identity of the residues that defined this RBD-ACE2 binding interface to perform a docking experiment to assess the ability of a heparin octasaccharides (octasaccharide taken from PDB 5UE2) to inhibit the interaction between RBD and ACE2. We hypothesized that the available polar sidechains of N487, Y489, Q493, Q498 and Y505 on the spike protein RBD along with other residues would bind to heparin and inhibit RBD-ACE2 interaction. Using the autodock software suite we performed an exhaustive search of various conformations that the glycosaminoglycan could sample at the ACE2 binding surface of the spike protein RBD. From this experiment we were able to identify a binding mode of the heparin octasaccharide that would restrict RBD-ACE2 binding. In this conformation the heparin octasaccharide forms a hydrogen bond network with N448, N450, Q493 and N501 that aids in its occupancy of this binding region and sterically restricts access to Q498, Y489 and Y505.

Supplementary Table S1. *In vitro* effects of sulfated polysaccharides on the inhibition of SARS-CoV2 infection.

Poly-saccharides	Mw[a] (kDa)	Anti-coagulant activity[b]	Scaffolds	Binding inhibition[c] (%)	EC_{50} (μg/mL)	EC_{50}[e] (μM)
NACH	4	-	Linear	ND[d]	221 ± 122	55
TriS-heparin	17.6	-	Linear	72 ± 8.0	88 ± 27	5.0
Heparin	17	+	Linear	82 ± 7.5	36 ± 14	2.1
RPI-27	100	-	Branched	97 ± 1.5	8.3 ± 4.6	0.08
RPI-28	12	-	Branched	90 ± 1.7	16 ± 11	1.2

[a]Average molecular weight.
[b]Antithrombin III-mediated anticoagulant activity
[c]Competitive binding inhibition between polysaccharide-S-protein complex and heparin (HP)-SPR chip.
[d]Not detected.
[e]The values obtained by dividing average molecular weight.

References

1 Matsuyama, S. *et al.* Enhanced isolation of SARS-CoV-2 by TMPRSS2-expressing cells. *Proc. Natl. Acad. Sci. USA* **117**, 7001-7003 (2020).

2 Harcourt, J. *et al.* Severe Acute Respiratory Syndrome Coronavirus 2 from Patient with Coronavirus Disease, United States. *Emerg. Infect. Dis.* **26**, 1266-1273 (2020).

3 Shang, J. *et al.* Structural basis of receptor recognition by SARS-CoV-2. *Nature* **581**, 221-224 (2020).
